# Editorial: Personalized nutrition with polyphenols—which, for whom, when and how?

**DOI:** 10.3389/fnut.2023.1193339

**Published:** 2023-05-04

**Authors:** Antje R. Weseler, Aalt Bast, Mireille M. J. P. E. Sthijns

**Affiliations:** ^1^Department of Medicine, Medical School Hamburg (MSH), Hamburg, Germany; ^2^FSE Campus Venlo, Faculty of Science and Engineering, Maastricht University, Venlo, Netherlands; ^3^Food Innovation and Health, Department of Human Biology, Faculty of Health, Medicine and Life Sciences, School of Nutrition and Translational Research in Metabolism (NUTRIM), Maastricht University, Venlo, Netherlands

**Keywords:** polyphenols, flavonoids, cardiovascular disease, inflammation, oxidative stress, personalized nutrition, individualized treatment, dietary supplements

Epidemiological data indicate that the regular consumption of diets rich in vegetables and fruits is associated with a reduced risk of chronic cardiometabolic and neurological diseases (1, 2). The high content of polyphenols has been associated with the health benefits of such plant-based diets because they exert a multitude of effects on cellular and molecular processes like inflammation, oxidative stress, energy metabolism, and cell proliferation (3). However, during the last 2 decades, randomized double-blind controlled dietary intervention studies could not unequivocally generate clear cause-effect relationships between the intake of such compounds and putative health effects in humans. The high variability between the individual subjects' responses to a polyphenolic intervention appears to be largely relevant to the inconsistency in study outcomes (4). Multiple personal characteristics have been identified to affect an individual's response like age, sex, comorbidities, medication, lifestyle, genome, epigenome, and the gut microbiome. However, the qualitative and quantitative impact of these person-specific features on an individual's response - regardless of whether it is to a drug with a single chemical agent or to a plant extract or meal consisting of a complex mixture of countless phytochemicals - is still unknown.

In the current Research Topic, we aimed at collecting studies that contribute to further elucidate how the intake of polyphenols (which?) can be more targeted to an individual's need (for whom and when?) and how the health benefits for an individual can be assessed then.

## Which polyphenols are suitable for whom and when?

The notion, that polyphenols are not a panacea for everyone in every life situation but may also exert unintended effects on an individual‘s health already at an extremely early stage of life becomes evident from a mice study by Godschalk et al.. A genistein-rich diet during pregnancy results in increased levels of oxidative damage in testes and sperms of male off-springs, in particular when the antioxidant defense system of their mothers is (genetically) compromised. Although the relevance of these findings to humans needs to be demonstrated yet, it illustrates how specific polyphenols, i.e., the isoflavone genistein, ingested by women during a particular phase of life, i.e., pregnancy, may affect the health of individuals of subsequent generations.

Despite the lack of translatability, data from mouse models of complex diseases like high-fat- induced obesity and liver steatosis could hint at patients who may benefit from a targeted supply of particular dietary agents (Zhang et al.). At the same time, novel molecular mechanisms and biomarkers may be found capturing the multitude of physiological effects of a complex plant extract in a living organism.

## How can health effects be measured on an individual's level?

To assess person-specific responses to a particular intervention and to quantify inter- and intra-person variabilities in outcome parameters linked to a person‘s health or disease state new research methods are required. Recently, “single subject” or “n-of-1” studies have been advocated as a highly suitable approach to measure the health benefits of nutritional interventions for an individual (5).

Bapir et al. demonstrate how such an “n-of-1” study can be executed. By a series of double-blind randomized placebo-controlled cross-over n-of-1 trials, they measured the effects of a single cocoa flavanol consumption on blood pressure, heart rate, and pulse wave velocity (PWV) in 11 young healthy volunteers under real-life conditions with personal devices. The findings uncover the typical high variability between the subjects‘ blood pressure and PWV responses upon the single intake of a relatively high dose (ca. 860 mg) of cocoa flavanols. At the same time, a considerable day-to-day variation in a person‘s response to the flavanol intake becomes visible depending on their baseline blood pressure on that day. The use of home blood pressure monitoring devices is a pragmatic way to collect real life data of a person which will certainly be eased in the future by the availability of smartphones/-watches that can monitor multiple health parameters with increased accuracy.

Daily clinical practice is persistently challenged to identify people with an increased disease risk and initiate tailor-made prevention strategies as early as possible. Using oral glucose tolerance test (OGTT) data routinely ordered by physicians for a large cohort of individuals (*n* = 1198) (Olivera-Nappa et al.) established a mathematical 5- compartment model. This model allows capturing already subtle modulations in the dynamics of an individual‘s glycemic and insulinemic response way before conventional diagnostic parameters would pick up such changes. Subsequently, based on repetitions of a routine OGTT and using this model physicians can monitor an individual‘s response to e.g., a targeted supply of dietary agents like polyphenols over time.

Measures allowing to capture already subtle effects of changes in diet and physical activity are urgently required, because they improve not only an individual‘s health but also the financial situation of health care systems by the prevention of expensive pharmacological or surgical treatments.

In this respect, a thorough analysis of molecular pathways of crucial (patho)physiological processes in the human body like acute inflammation and its resolution during the healing process, also helps to identify new biomarkers for this purpose (Sears and Saha). In how far though these 3 suggested blood markers, i.e., the ratio of triglycerides to HDL cholesterol, the ratio of eicosapentaenoic acid to arachidonic acid, and the glycated hemoglobin HbA1c levels, are sensitive enough to detect the subtle effects of nutrients in a person remains to be shown yet by appropriate clinical data.

## What can we conclude for the future?

As in the medical field, also in nutrition research awareness is increasing that a “one-size-fits- all” approach for polyphenolic supplementations will not necessarily improve an individual‘s health. The various papers submitted here add from different perspectives valuable pieces to the big puzzle of how “personalized nutrition with polyphenols” can be investigated and implemented as an effective strategy to prevent diseases and improve the health status of an individual in daily life.

Without a doubt, the systematic development and application of personalized treatment approaches with both, drugs and dietary compounds, are exciting future tasks that will require out-of-the-box thinking and courage to break new ground in research and clinical application strategies.

## Author contributions

AW wrote the editorial. All authors revised the manuscript and approved its publication.
